# Successful treatment of HIV‐associated lupus‐like glomerulonephritis with mycophenolic acid

**DOI:** 10.1002/ccr3.2955

**Published:** 2020-05-19

**Authors:** Mark Tiong, Scott Wilson, Alan Pham, Anastasia Chrysostomou

**Affiliations:** ^1^ Department of Renal Medicine Alfred Health Melbourne Vic. Australia; ^2^ Department of Nephrology The Royal Melbourne Hospital Parkville Vic. Australia; ^3^ Department of Medicine Monash University Melbourne Vic. Australia; ^4^ Department of Medicine Epworth HealthCare Melbourne Vic. Australia; ^5^ Department of Anatomical Pathology Alfred Health Melbourne Vic. Australia

**Keywords:** HIV Immune Complex Kidney disease, HIV‐associated kidney disease, Human immunodeficiency virus, Mycophenolic acid

## Abstract

HIV‐associated lupus‐like glomerulonephritis is an uncommon but well‐described entity. Treatment has traditionally focused on control of HIV viremia with some using adjuvant steroids. Mycophenolic acid may prove to be a novel, nonsteroid, therapy in patients with active glomerulonephritis despite control of the underlying infection.

## Graphical abstract

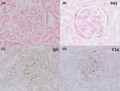

## 1 INTRODUCTION

A 34‐year‐old HIV‐positive man was diagnosed with HIV‐associated lupus‐like glomerulonephritis. The patient's retrovirus was already well‐controlled on combination antiretrovirals. The glomerulonephritis was treated with mycophenolic acid with excellent response. We consider the potential role of mycophenolic acid as a novel therapy for this increasingly recognized entity.

In the era of combination antiretroviral therapy (cART), HIV‐associated glomerulonephritis is an uncommon cause of renal disease in HIV‐positive individuals. There are limited data to guide treatment, but antiretroviral therapy, targeted to achieve a reduction in retroviral burden and normalization of CD4 T cell counts, renin‐angiotensin system blockade and systemic corticosteroids have previously been used. Given the metabolic consequences of systemic corticosteroids and the background significant burden of cardiovascular and metabolic disease in the HIV population, well‐tolerated steroid‐sparing or steroid‐avoiding regimens are likely more desirable where renal disease persists despite suppression of detectable circulating virus.

## 2 CASE REPORT

A 34‐year‐old HIV‐positive Caucasian man presented with recurrent episodes of fever, myalgia, and macroscopic hematuria. Each episode was self‐resolving, typically lasting 1‐3 days. Investigations, including urinalysis and serum creatinine, had been previously unremarkable in between clinical episodes.

HIV infection had been diagnosed 12 years prior to the current presentation, predating the earliest onset of febrile hematuric episodes by approximately 2 years. Circulating retroviral load was persistently below the detectable threshold by polymerase chain reaction since commencing cART 5 years prior. The decision to commence cART had been made in accordance with the evolution of international HIV treatment guidelines rather than any clinical or hematological indication.^1^

The febrile episodes started approximately 10 years prior and typically occurred 2‐3 times per year, though more recently had increased to once every 1‐2 months, which prompted presentation for assessment. There was a significant decrement in renal function noted with a serum creatinine of 160 μmol/L (eGFR 45 mL/min/1.73 m^2^), from 94 μmol/L (eGFR 90 mL/min/1.73m^2^) one year prior. Investigation during an acute febrile episode demonstrated proteinuria (uPCR 80 mg/mmol) with hematuria (urinary erythrocytes > 1000 × 10^6/L) of glomerular morphology. Testing for co‐existent infections including hepatitis B and C, Syphilis, gonorrhea, chlamydia, Brucella, Rickettsia, Q fever, strongyloides, mycobacterium tuberculosis, parvovirus, and malaria were all negative, as was screening for familial Mediterranean fever and porphyria. There was serological evidence of previous EBV and CMV exposure, but not of active or recent infection. There was no other past medical history of note, including no history of diabetes mellitus or hypertension.

**Figure 1:**
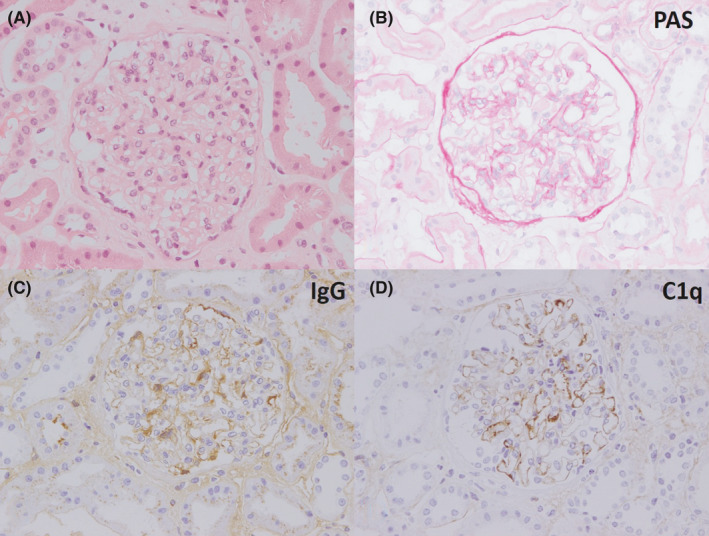
Renal biopsy showing “lupus like” changes. Mild increase in glomerular mesangial cellularity on hematoxylin and eosin stain (A) and PAS stain (B), with glomerular capillary and mesangial positivity for IgG (C) and C1q (D) on immunoperoxidase staining

A renal biopsy was obtained showing renal cortex without any interstitial fibrosis or interstitial inflammation (Figure 1). A total of 36 glomeruli were present, none of which were globally sclerosed. By light microscopy, glomeruli showed a focal (10 glomeruli) mild segmental increase in mesangial cellularity. There was no endocapillary proliferation, no segmental sclerosis, no wire loops, no basement membrane spiking or hyaline thrombi. There were no crescents or necrotizing lesions. Immunoperoxidase stains showed granular capillary basement membrane staining for IgA, IgG, IgM, C3, and C1q with weak focal mesangial staining for IgA, IgG, and C1q. Electron microscopy was not performed.

Concurrent serological testing was negative for antinuclear antibodies, anti‐Smith, and anti‐double stranded DNA. Serum levels of C3 and C4 were within the normal laboratory reference range, and rheumatoid factor was not detectable. Given his history of HIV positivity and negative systemic lupus erythematosus (SLE) serology, a diagnosis of HIV‐associated lupus‐like glomerulonephritis was made.

Multiple treatment options including active surveillance and systemic corticosteroids were considered and discussed with the patient. There was a general reluctance expressed by the patient to utilize systemic corticosteroids, due to perceived burden of adverse effects. Given an established role in the treatment of LN and favorable side effect profile, a trial of mycophenolic acid (MPA) was also offered. Following informed consent, 1 gram of oral mycophenolate mofetil was commenced twice a day. Since commencing MPA the patient has experienced a dramatic reduction in frequency of febrile hematuric episodes, with only a single event now 12 months into treatment. Biochemical renal function has also returned to the previous baseline with a serum creatinine of 90 μmol/L (eGFR 90 mL/min/1.73m^2^).

## 3 DISCUSSION

HIV‐associated lupus‐like glomerulonephritis is a rare, but well‐recognized cause of renal disease in HIV‐positive individuals. It is commonly included on the spectrum of “HIV Immune Complex Kidney disease” (HIVICK) though whether this heterogenous group represents distinct disease processes remains unclear.^2^

HIV‐associated lupus‐like glomerulonephritis is described in patients with renal biopsy features which are “lupus‐like” both histologically and by immunofluorescence markers, but occur in HIV‐infected patients who otherwise lack serological and clinical evidence of SLE.^3^ Patients with primary LN with concurrent HIV infection are also acknowledged. While glomerular immune deposits may be analyzed for the presence of HIV antigens, this is not generally available outside of the research setting.^2^ This, and the lack of fully objective criteria for primary SLE, creates the possibility of diagnostic misclassification.^4^ Our patient did not meet conventional diagnostic criteria for SLE,^5^, ^6^ and while seronegative SLE represents a potential differential, this entity is also exceedingly rare.^5^, ^6^, ^7^ Episodic fever was a prominent feature of our patient's presentation, and while not universal of this disease entity, has been previously described.^8^ Importantly, extensive testing for concurrent infection was persistently negative, as was other markers of autoimmune disorders. While there was limited testing for periodic fever syndromes (the patient tested negative for familial Mediterranean fever), these entities would not account for the renal histopathological findings.^9^ Given the lack of alternative explanation and the presence of established HIV infection, which preceded the initial presentation of febrile hematruic episodes, the patient's renal pathology was attributed to consequence of HIV rather than an alternative process.^10^

Beyond empiric recommendations for renin‐angiotensin system inhibition, there is limited data to guide treatment of patients with either HIV‐associated lupus‐like glomerulonephritis, or HIVICK more broadly.^2^, ^11^ There is an apparent protective association between cART use and reduced risk of HIVICK, with cases typically arising where there is an established history of untreated HIV infection or suppressed CD4 T lymphocyte count. Multiple case reports, generally from the era preceding the advocacy of universal cART upon HIV diagnosis, have suggested benefit from starting cART in untreated HIV patients with lupus‐like renal disease.^12^, ^13^, ^14^ Our patient was already established on cART with his retroviral infection well‐controlled. Mechanistically, we postulated that despite the absence of detectable HIV viremia, there was ongoing sequalae attributable to the initial immunostimulatory event, manifesting as ongoing fevers and active glomerulonephritis. This is perhaps analogous to the subgroup of patients with hepatitis‐C‐associated cryoglobinemic vasculitis, who have ongoing glomerular disease despite successful treatment of the causative hepatitis infection. In such patients, treatment with immunomodulatory therapies such as rituximab is now recommended in international guidelines.^15^

The role of immunosuppression in HIVICK has a limited clinical evidence base. Experience with corticosteroids in HIVAN has often been extrapolated and used as a rationale for therapeutic trials of systemic corticosteroids in individual cases of HIVICK, including in several cases of lupus‐like glomerulonephritis, with some benefit.^2^, ^8^, ^11^, ^16^, ^17^ The progressive frequency of febrile hematuria and significant loss of renal function was considered the indication for specific immunomodulatory therapy in this case. Patients with HIV infection are at increased risk of metabolic and cardiovascular disease, making the potential adverse effects of systemic corticosteroids undesirable.^18^ Our patient was reluctant to be treated with corticosteroids which prompted consideration of alternative immune‐modulating agents.

MPA has become the standard of care for induction and maintenance therapy in proliferative lupus nephritis with a favorable efficacy and tolerability profile.^19^ Active LN is broadly characterized by reactive inflammation to immune complex deposition in the glomeruli. MPA appears to modulate this response, thereby reducing inflammatory‐mediated kidney injury.^19^ Through inhibition of inosine‐5’‐monophosphate dehydrogenase, guanosine nucleotides in lymphocytes are depleted, suppressing normal T‐ and B‐cell proliferation pathways.^20^

In HIVICK, circulating HIV antigen immune complexes have been found deposited in the kidneys.^21^ Similar to LN, these circulating complexes are thought to trigger immune‐mediated inflammatory kidney damage, which may explain why patients with HIV‐associated lupus‐like glomerulonephritis have histopathological lesions that closely resemble those of LN. These observations led us to postulate that our patient may be responsive to therapies traditionally used in primary LN. The safety of using cytostatic immunosuppressive medications in patients with HIV infection is an important consideration, as is potential interactions with cART. MPA has been used successfully in HIV‐positive renal transplant recipients, as an adjunct antiviral, and in other novel cases, where HIV is thought to act as an immunostimulatory trigger, without apparent deleterious effect in terms of retroviral control, excess adverse events or opportunistic infections.^22^, ^23^, ^24^

Since commencing MPA therapy, there has been a clear demarcation in the trajectory of his clinical episodes with normalization of biochemical renal function and urinalysis—a signal that immunomodulation is favorably altering the underlying disease process.

## 4 CONCLUSION

While further data to confirm efficacy and safety is needed, mycophenolic acid may be an effective novel treatment option in patients with HIV‐associated lupus‐like glomerulonephritis, particularly in those who have not responded to, or are already treated with cART.

## CONFLICT OF INTEREST

None declared.

## AUTHOR CONTRIBUTIONS

MT: initiated the preparation of this manuscript. All authors were involved in the clinical management of this patient and contributed to the preparation of this manuscript.

## RESEARCH INVOLVING HUMAN PARTICIPANTS AND/OR ANIMALS

This article does not contain any studies with human participants performed by any of the authors.

## INFORMED CONSENT

Informed consent was obtained from all individual participants included in the study.
